# Collapsin response mediator protein 4 promotor methylation level as a potential predictor for diagnosing primary malignant lymphoma of the prostate

**DOI:** 10.1186/s12935-017-0484-9

**Published:** 2018-01-04

**Authors:** Zheng Chen, Qiong Liang, Jue Wang, Qun-Xiong Huang, Jian-ning Chen, Zi-jin Weng, Chun-kui Shao, Xin Gao, Jun Pang

**Affiliations:** 1 Department of Urology, The Third Affiliated Hospital, Sun Yat-sen University, Tianhe Road 600, Guangzhou, 510630 China; 2 Department of Pathology, The Third Affiliated Hospital, Sun Yat-sen University, Guangzhou, China; 3 Department of Pathology, The First Affiliated Hospital, Sun Yat-sen University, Guangzhou, China

**Keywords:** Primary malignant lymphoma prostate (PMLP), Collapsin response mediator protein 4 (CRMP4), Methylation, ROC curve, Predictor

## Abstract

**Background:**

Primary malignant lymphoma of the prostate (PMLP) is prone to occur in the elderly, and it has no significant correlation with lactate dehydrogenase (LDH) and prostate specific antigen (PSA). Clinical symptoms and imaging data of PMLP remain unspecific, and its prognosis is poor. A previous result showed that collapsin response mediator protein 4 (CRMP4) promotor methylation can be used as a predictor for lymph node metastases in prostate biopsies. However, the relationship between CRMP4 promotor methylation and PMLP has not been studied.

**Methods:**

We investigated the clinicopathological features of PMLP and the significance of CRMP4 methylation in PMLP. The clinical data and diagnosis information of 10 patients with PMLP were retrospectively analyzed. The CRMP4 promotor methylation level in paraffin-embedded tissues of the 10 patients with PMLP were determined and then compared to limited prostate cancer (LPCa) and its negative lymph node tissue [LPCa-LN (−) (10 cases)] and also to metastatic prostate adenocarcinoma (mPCa) and its positive lymph node tissue [mPCa-LN (+) (10 cases)]. Methylation of the CRMP4 promotor in each group was analyzed statistically. A receiver operating characteristic (ROC) curve was used to analyze the diagnostic value of CRMP4 methylation in PMLP.

**Results:**

The average methylation value of CRMP4 in 10 PMLP patients, 20 cases of prostate adenocarcinoma tissue, 10 cases LPCa-LN (−) and 10 cases mPCa-LN (+) were 42.3, 30.6, 6.7 and 20.3%, respectively. A Kruskal–Wallis test showed that the difference of CRMP4 methylation was significant (X^2^ = 38.0, P < 0.001). An ROC curve analysis found that CRMP4 methylation > 40.9% could diagnose PMLP. This method had 90% sensitivity and 95% specificity under conditions of CRMP4 methylation > 40.9%. The area under the curve (AUC) was 0.957.

**Conclusions:**

Methylation of the CRMP4 gene was significantly increased in PMLP, and it is expected to become a new predictor for PMLP.

## Background

Primary malignant lymphoma of the prostate (PMLP) is a rare prostatic malignancy [1, 2]. PMLP includes Hodgkin’s lymphoma (HL) and non-Hodgkin’s lymphoma (NHL) according to the pathological features, of which NHL is more common [3–5]. The incidence of NHL is approximately 1‰, the majority of which are of the diffuse large B cell (DLBCL) NHL subtype [4, 6, 7]. Although there is a low incidence of lymphoma involving the prostate gland, it needs further research to accurately diagnose. Currently, most reports detail information about only one or two PMLP cases [5, 8, 9]. The Mayo Clinic and Hopkins Hospital have retrospectively analyzed 7 cases of PMLP over 115 years [10]. Previous results showed that altered expression levels of collapsing response mediator proteins (CRMPs) (CRMP1-5) are associated with several malignant tumors, including lung, breast, colorectal and prostate cancer [11]. Of all CRMP family members, the CRMP4 gene is the only gene differentially expressed in prostate cancer tissues [11, 12]. CRMP4 was first discovered and reported as a PCa transfer suppressor gene by our research team during a proteomics screen of proteins related to prostate cancer (PCa) metastasis [12]. However, the role of CRMP4 methylation in PMLP has not yet been studied.

This study collected 10 cases of PMLP from November 2006 to December 2016 at Sun Yat-sen University and analyzed the clinicopathological features and diagnosis of PMLP, further increasing our understanding of the disease. At the same time, CRMP4 methylation was detected in paraffin tissue samples of these patients, and the significance of CRMP4 methylation in PMLP was determined.

## Materials and methods

### Clinical data

Ten cases of PMLP patients with age of onset from 57 to 82 years old and a median age of 69 years old were treated in the Third Affiliated Hospital and the First Hospital of Sun Yat-sen University during November 2006 to December 2016. All patients had lower urinary tract obstruction symptoms, including urinary frequency, urgency, nocturia increased (more than four times/night) and dysuria, 4 cases of urinary retention, and 2 cases with hematuria. All patients were without fever, night sweats, weight loss or other lymph tumor symptoms. The prostates from the 10 cases had different degrees of hyperplasia and disappearance of the central ditch via transrectal ultrasound and palpation. There were 3 cases of palpable prostate nodules among the 10 cases, including 1 case of palpable prostate nodules with a hard texture. Secondary PMLP was excluded by preoperative computed tomography (CT) or nuclear magnetic resonance imaging (MRI) examination [13]. Additionally, postoperative bone marrow biopsy excluded outer lymphoma involvement. All 10 patients underwent surgical treatment [6 cases of transurethral resection of the prostate (TURP) surgery and 4 cases of laparoscopic radical prostatectomy (LRP)].

### Hematoxylin and eosin (H&E) stain and immunohistochemistry (IHC)

Ten patients were subjected to conventional H&E preparation and immunohistochemical staining. Microscopy was performed by two pathology doctors (WJ and JNC) according to the 2008 WHO classification of lymphoma neoplasms, and then two senior doctors (QL and CKS) rechecked the microscopy and made the final diagnosis. Antibodies CK, LCA, CD3, CD45RO, CD20, CD79a, CD5, Cyclin D1, CD10, MUM1, Bcl2, Bcl6, CD30, and Ki-67 were obtained from Abcam USA and DAKO Denmark. The processes were as follows: immunohistochemical staining for 4-μm tissues were performed with anti-CK (1:100), anti-LCA (1:400), anti-CD3 (1:100), anti-CD45RO (1:500), anti-CD20 (1:250), anti-CD79a (1:100), anti-CD5 (1:100), anti-Cyclin D1 (RTU), anti-CD10 (1:50), anti-MUM1 (1:50), anti-Bcl2 (1:100), anti-Bcl6 (1:100), anti-CD30 (1:40), and anti-Ki-67 (1:100) and were detected using the DAKO En-Vision System (Dako Diagnostics, Zug, Switzerland) according to the manufacturer’s instructions.

### Specimen collection, DNA extraction and tumor specimen preparation

The 10 paraffin-embedded tissues of PMLP were collected. Additionally, 10 specimens of paraffin-embedded tissue and negative lymph nodes were collected from 10 patients with localized prostate adenocarcinoma, and another 10 specimens of paraffin-embedded tissue and positive lymph nodes were collected from 10 patients with metastatic prostate adenocarcinoma. Each specimen was cut into 10 slices of 5 μm. Prostate samples were collected from men who were enrolled in the study and eligible for inclusion based on the abovementioned criteria. All cases underwent a central pathological review before CRMP4 promoter methylation analysis was performed. After dewaxing, DNA was extracted using a DNA extraction kit Qiagen (# 69506) according to the manufacturer’s instruction.

### Sodium bisulfite modification of DNA

Bisulfite modification was performed by a bisulfite treatment kit (EZ DNA Methylation-Gold™ Kit) according to the manufacturer’s instruction.

### Methylation detection

Polymerase chain reaction (PCR) primers (Table 1, sequence labeled F and R) were designed for two CpG islands around the CRMP4 promoter. Sequencing primers are also presented in Table 1 (sequence labeled S). Six pairs of specific methylation PCR primers (S1–S6) were designed around the different methylation site promoter CpG islands. PCR amplification of pyrophosphoric acid sequencing templates was performed. Then, the PCR products and Streptavidin Sepharose HP beads were mixed and incubated for pyrosequencing [Pyromark ID96 system (Biotage, Uppsala, Sweden) with the instrument software (PyroMark CpG Software V1.0.11)].

**Table 1:** PCR primers of CRMP4 promoter and sequencing primers

CPG1	
CZ-G1-F1	ATAGGGAATGGTTGAGTTTATTGT
CZ-G1-R1-Bio	Biotin-ACCCCCTCTCCTCTACCATA
CZ-G1-S1	GTTTTTTGTAGTTTTTGAGA
CZ-G1-S2	AGTTTTTTTTTAGAATAAAG
CZ-G1-S3	TTTAGGAGTTTGAAGGG
CZ-G1-S4	GTTTAGGGTTTGGGGA
CZ-G1-S5	GGGAATGGTTGAGTTTA
CPG2	
CZ-G2-F2	AGGGTTTGGGGATTTTAGTAGGT
CZ-G2-R2-Bio	Biotin-TCCCCAAAATAAAAACATCAACT
CZ-G2-S6	TAGAGTTATGGTAGAGGAGAG

### Statistical methods

Multiple comparisons between the two groups were performed by Dwass–Steel–Critchlow–Fligner test. P < 0.05 was statistically significant. All statistical analyses were performed using statistical software SAS9.3. The ROC curve analysis was performed using Medical version 12 statistical software.

## Results

### Clinical results

The average prostate specific antigen (PSA) of PMLP patients was 2.8 ng/ml (0.54–4.82 ng/ml), which was lower than that of Pca with LN (−) (8.86–38.46 ng/ml) and PCa with LN (+) (10.07–87.22). The average serum lactate dehydrogenase (LDH) was 320.4 (153–560) U/l, which was above the normal upper limit by 7/10 (70%) (Table 2).

**Table 2:** Clinicopathological characteristics of the patients

Items	PMLP	PCa with LN (−)	PCa with LN (+)
No. of cases	10	10	10
Median age (y, range)	69 (57–82)	67 (46–79)	68 (52–83)
Mean PSA (ng/ml, range)	2.8 (0.54–4.82)	12.63 (8.86–38.46)	16.86 (10.07–87.22)
Mean LDH (U/l, range)	320.4 (153–560)	–	–
Biopsy Gleason sum n			
≤ 6	–	3	1
3 + 4	–	3	3
4 + 3	–	2	3
8–10	–	2	3
Pathological Gleason sum n			
≤ 6	–	2	0
3 + 4	–	2	2
4 + 3	–	3	4
8–10	–	3	4
Pathological T stage n			
T2a	0	2	0
T2b	4	2	0
T2c	5	5	0
≥ T3a	1	1	10
Lymph node invasion n			
Negative	10	10	0
Positive	0	0	10

### CT or MRI

**Fig. 1:**
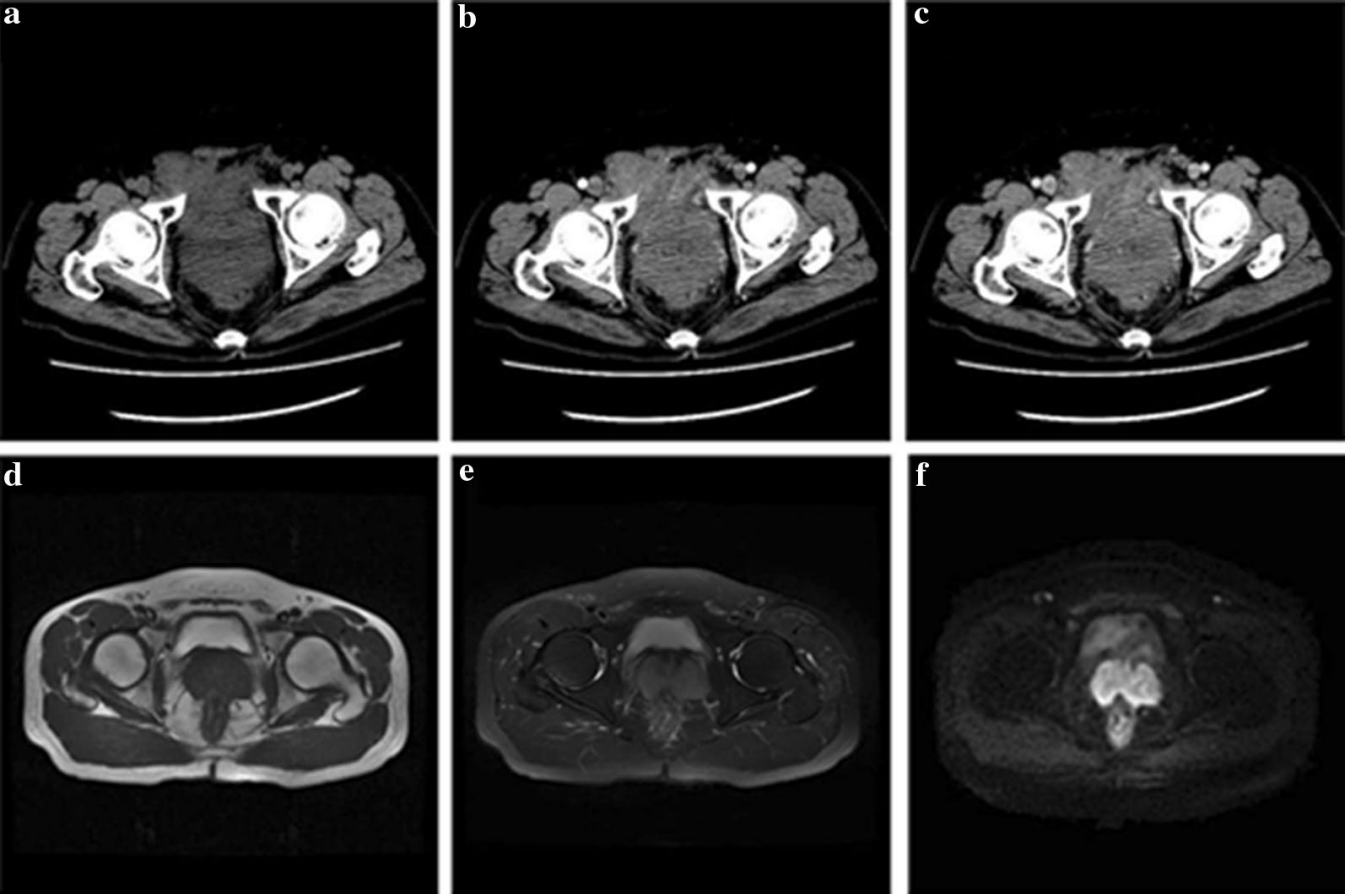
Preoperative diagnosis was performed by pelvic CT or MRI examination. The results suggested that the prostate was significantly augmented, the internal density of the prostate was uneven and spot-like calcified. Pelvic CT figure: **a** plane scan, **b** curve arterial phase, **c** curve vein; MRI figure: **d** T1W1, **e** T2W1, **f** DWI

To exclude secondary prostate lymphoma, preoperative diagnosis was performed by CT or MRI examination. PMLP diagnostic criteria recommended by Bostwick and Mann were as follows: (1) presenting symptoms attributable to prostatic enlargement; (2) involvement of the prostate predominantly, with or without involvement of adjacent tissue; and (3) absence of involvement of the liver, spleen or lymph nodes after at least 1 month of diagnosis of the prostatic involvement [1, 10]. According to the results of the CT and MRI examinations as well as the PMLP diagnostic criteria, the 10 patients were confirmed PMLP (Fig. 1).

### Pathological results

**Fig. 2:**
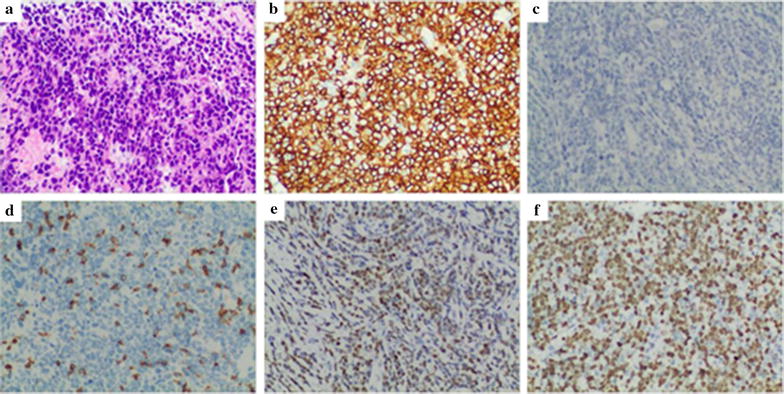
IHC results of DLBCL. **a** Hematoxylin and eosin (H&E) stain, ×200. **b** CD20 (positive), IHC, ×200, **c** CD10 (negative), IHC, ×200. **d** CD3 (background small lymphocytes positive, tumor cell negative). IHC, ×200. **e** MUM1 (positive), IHC, ×200; **f** Ki-67 (70% +), IHC, ×200

**Fig. 3:**
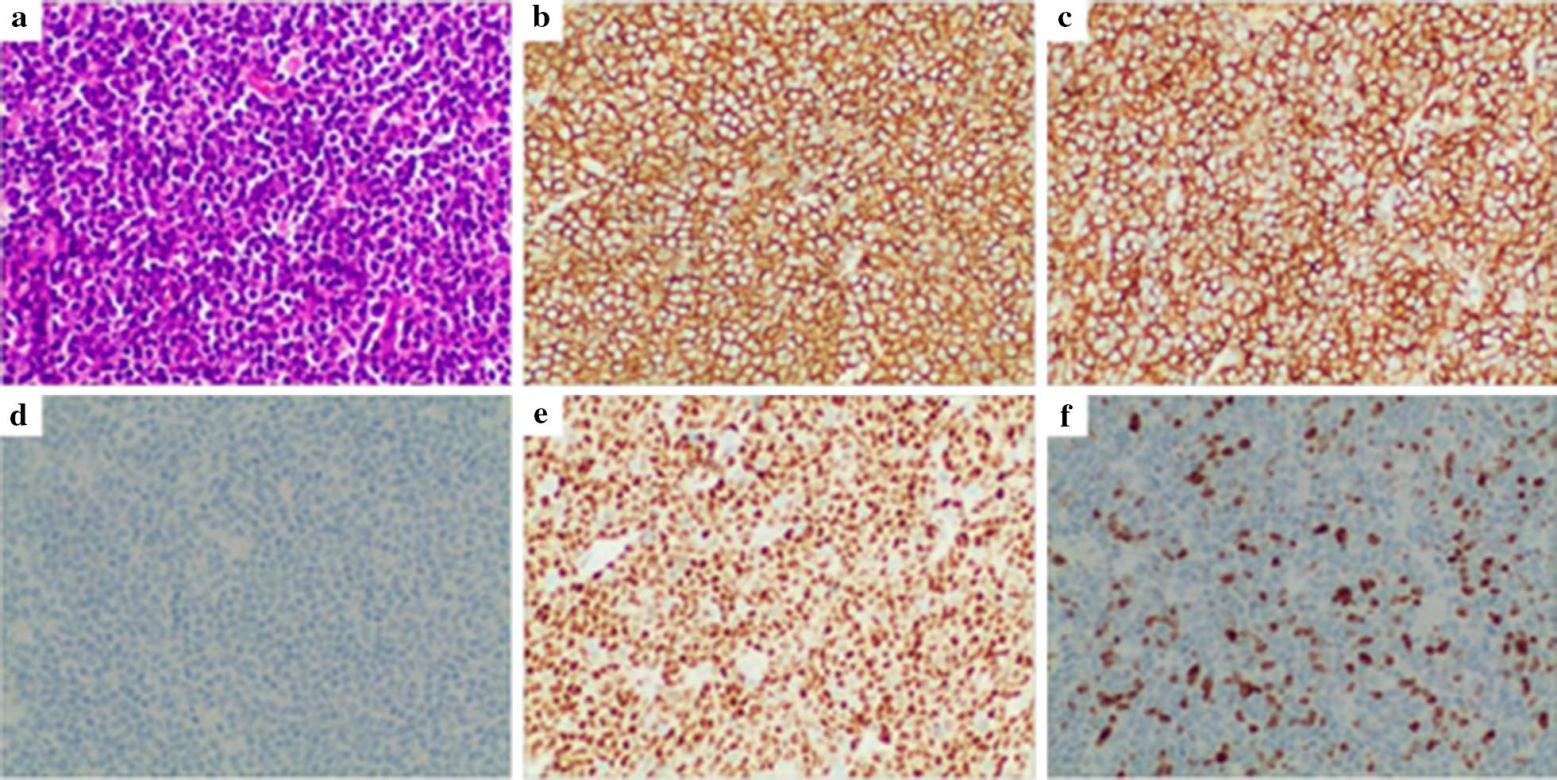
IHC results of MCL. **a** H&E stain, ×200. **b** CD20 (positive), IHC, ×200. **c** CD5 (negative), IHC, ×200. **d** CD10 (negative), IHC, ×200. **e** Cyclin D1 (positive), IHC, ×200; **f** Ki-67 (30% +), IHC, ×200

Immunohistochemistry results of 10 patients were shown in Table 3. All cases were as follows: epithelial marker CK (−); T cells labeled CD3 or CD45RO (−); and B cells labeled CD20 and CD79a diffuse (+). All cases were B cell-derived NHL (n = 10), including 9 cases with diffuse large B cell lymphoma (DLBCL) and 1 case of mantle-cell lymphoma (MCL). The sample size of prostate adenocarcinoma in PMLP was enlarged. The morphological features of 9 cases DLBCL were as follows: destruction of the normal structure of the prostate; large lymphocyte infiltration; round, oval or vacuole-like nucleus; finer chromatin; visible center nucleolus or 2–4 nucleoli near the nucleus membrane; less cytoplasm, biphasic or basophilic; and visibly mitotic. The background had a few small lymphocytes scattered. IHC showed that B cells were labeled CD20 and CD79a diffuse (+), of which 5 cases marked CD10 (+) or Bcl6 (+)/MUM1 (−) and 4 cases marked CD10 (−) and MUM1 (+). Observing the prostate of 1 case of MCL found that secondary small lymphocytes had a blurred nodular distribution, single cell morphology, irregular nucleus, nucleolus was not obvious, mitosis was visible, cytoplasm was scarce, and more transparent degeneration of small blood vessels was visible, and the IHC showed B cell markers CD20, CD79a, CD5, and CyclinD1 diffuse (+) (Figs. 2, 3).

**Table 3:** Nine cases of PMLP immunohistochemical information

	Surgical procedures	Pathological types	Immunophenotype
1	TURP	DLBCL	CK(−), LCA(+), CD45RO(−), CD20(+), CD79a(+), CyclinD1(−), CD10(−), MUM1(+), Bcl2(+), Bcl6(+), CD30(−), Ki-67(70% +)
2	TURP	DLBCL	CK(−), LCA(+), CD3(−), CD20(+), CD79a(+), CD5(−), CyclinD1(−), CD10(−), MUM1(+), Bcl2(+), Bcl6(+), Ki-67(55% +)
3	LRP	DLBCL	CK(−), LCA(+), CD3(−), CD45RO(−), CD20(+), CD79a(+), CyclinD1(−), CD10(+), CD30(−), Ki-67(40%)
4	TURP	MCL	CK(−), LCA(+), CD3(−), CD20(+), CD79a(+), CD5(+), CyclinD1(+), CD10(−), MUM1(+), Bcl2(+), Bcl6(−), CD30(−), Ki-67(30%)
5	LRP	DLBCL	CK(−), LCA(+), CD3(−), CD20(−), CD79a(+), CD5(−), CyclinD1(−), CD10(−), MUM1(−), Bcl2(+), Bcl6(+), CD30(−), Ki-67(90% +)
6	TURP	DLBCL	CK(−), LCA(+), CD45RO(−), CD20(+), CD79a(+), CD5(−), CyclinD1(−), CD10(−), MUM1(+), Bcl2(+), Ki-67(80% +)
7	TURP	DLBCL	CK(−), LCA(+), CD45RO(−), CD20(+), CD79a(+), CyclinD1(−), CD10(+), MUM1(−), Bcl2(+), Ki-67(80% +)
8	LRP	DLBCL	CK(−), LCA(+), CD45RO(−), CD20(−), CD79a(+), CD5(−), CyclinD1(−), CD10(+), MUM1(−), Bcl2(+), Bcl6(+), Ki-67(45% +)
9	TURP	DLBCL	CK(−), LCA(+), CD3(−), CD20(+), CD79a(+), CD5(−), CyclinD1(−), CD10(−), MUM1(−), Bcl2(+), Bcl6(+), CD30(−), Ki-67(80% +)
10	LRP	DLBCL	CK(−), LCA(+), CD45RO(−), CD20(−), CD79a(+), CD5(−), CyclinD1(−), CD10(−), MUM1(+), Bcl2(+), Bcl6(−), Ki-67(35% +)

### CRMP4 methylation in the promotor region

**Fig. 4:**
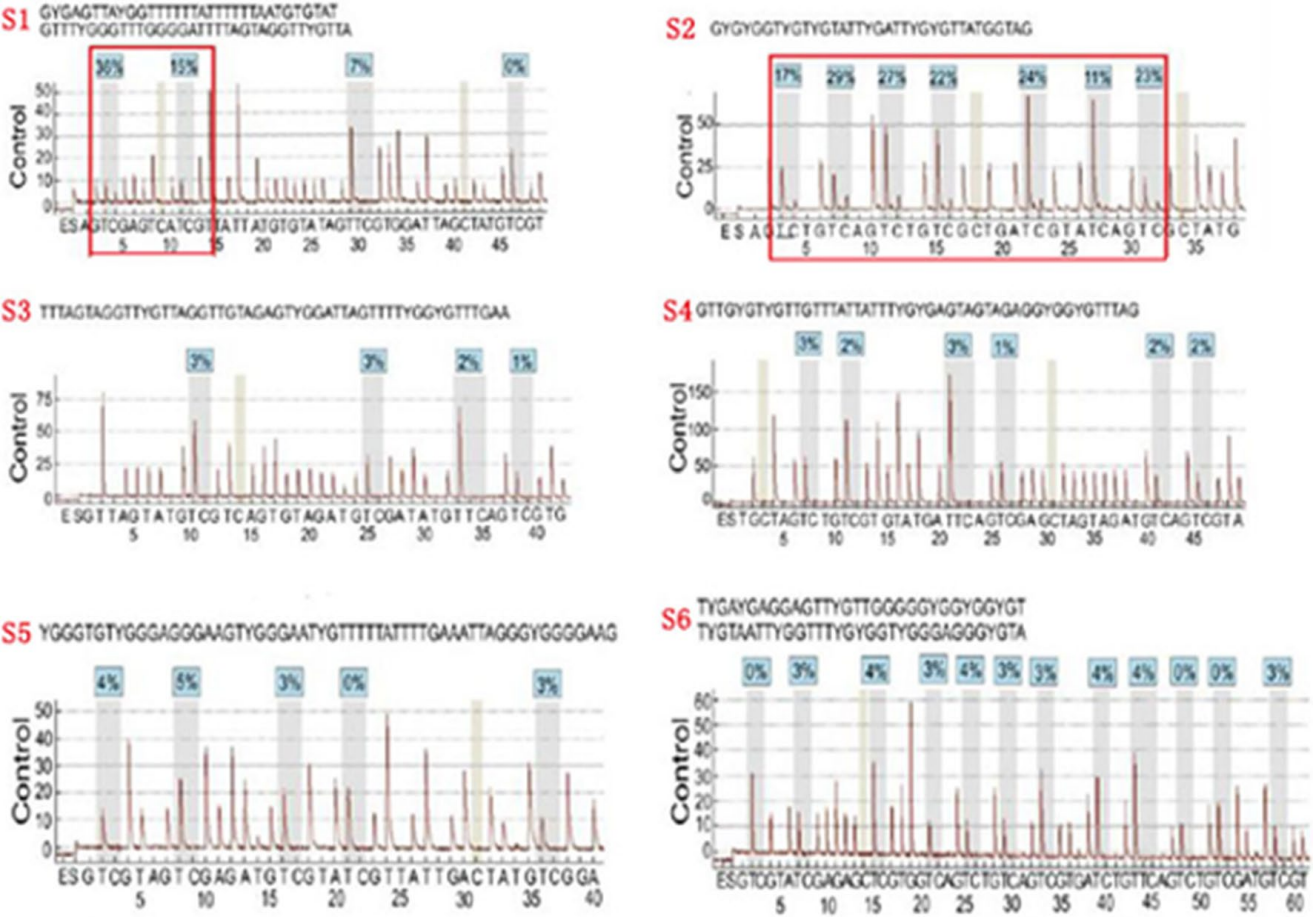
Quantitative pyrosequencing of methylation sites within CRMP4 promoter region. The CRMP4 gene promoter region A S1 (methylation sites − 848 and − 841) and region B S2 (methylation sites − 680, − 678, − 674, − 671, − 665, − 660, and − 658) was significantly methylated, and the methylation of site S3–S6 was not significant

**Fig. 5:**
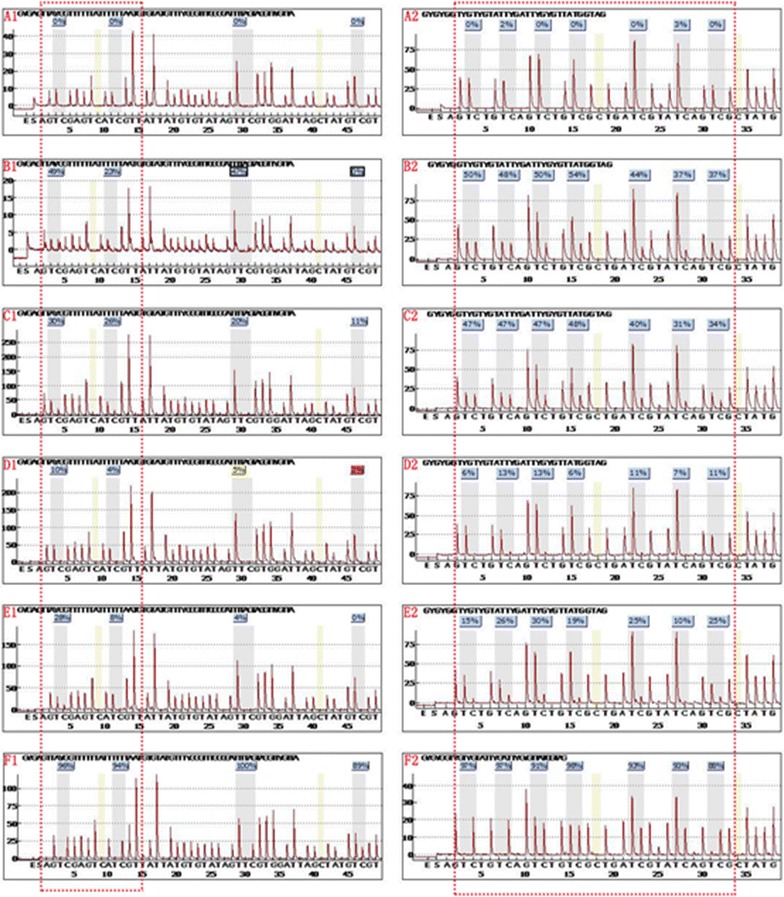
The CRMP4 methylation site was detected in each group. **A1**/**A2** methylation values of the unmethylated control group. **B1**/**B2** was histometric methylation values of 10 cases of PMLP tissue. **C1**/**C2** was histometric methylation values of 20 patients with prostate adenocarcinoma (10 patients with LPCa and 10 patients with mPCa). **D1**/**D2** was histometric methylation value of 10 cases of LPCa-LN (−). **E1**/**E2** was histometric methylation value of 10 cases of mPCa-LN (+). **F1**/**F2** was the methylation value of the control group. The red dotted frame represents S1 and S2 and is listed in the analyzed methylation site of CRMP4

**Fig. 6:**
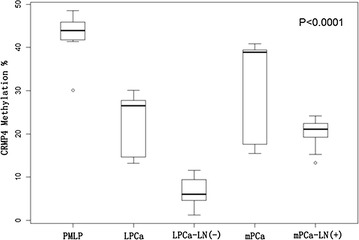
The box pattern shows the difference in CRMP4 methylation values for each group. The average methylation of CRMP4 promoter at nine sites (methylation sites − 680, − 678, − 674, − 671, − 665, − 660, − 658, − 848, and − 841) in PMLP, prostate adenocarcinoma (including LPCa and mPCa), LPCa-LN (−) and mPCa-LN (+) were 42.3, 30.6, 6.7, 20.3%, respectively. The difference was clear. *PMLP* Primary malignant lymphoma of the prostate, *LPCa* localized prostate cancer, *LPCa-LN* (*−*) negative lymph nodes of the localized prostate cancer, *mPCa* metastatic prostate cancer, and *mPCa-LN* (*+*) positive lymph nodes of the metastatic prostate cancer

The CRMP4 gene promoter region A S1 (methylation sites − 848, − 841) and region B S2 (methylation sites − 680, − 678, − 674, − 671, − 665, − 660, and − 658) were significantly methylated, and methylation of the site S3–S6 was not significant (Fig. 4). CRMP4 methylation in 10 cases PMLP, 20 cases of prostate adenocarcinoma (10 patients with localized PCa and 10 patients with metastatic PCa), 10 cases of negative lymph nodes of localized prostate adenocarcinoma (LPCa-LN (−)) and 10 cases of positive lymph nodes of metastatic prostate adenocarcinoma (mPCa-LN (+)) paraffin embedded tissues were determined. The results showed that the average methylation of the CRMP4 promoter occurred at 9 sites (methylation sites − 680, − 678, − 674, − 671, − 665, − 660, − 658, − 848, and − 841) in 10 cases of PMLP, 20 cases of prostate adenocarcinoma, 10 cases of LPCa-LN (−) and 10 cases of mPCa-LN (+) and were 42.3, 30.6, 6.7, 20.3%, respectively. The difference was clear (Figs. 5, 6).

### ROC curve analyzing the value of CRMP4 methylation in predicting PMLP

**Fig. 7:**
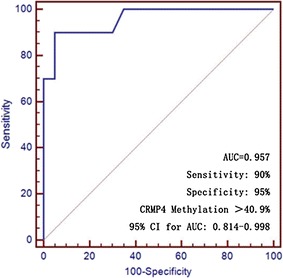
ROC curve analysis of predictive value of CRMP4 methylation in PMLP diagnosis. ROC curve analysis showed that the sensitivity of predicting PMLP was 90% when CRMP4 methylation > 40.9%. The predictive specificity was 95% and the area under curve (AUC) was 0.957

The CRMP4 methylation values for 10 prostatic tissues of PMLP patients and 20 cases of prostate adenocarcinoma were analyzed (Fig. 7). ROC curve analysis showed that the sensitivity of predicting PMLP was 90% when CRMP4 methylation > 40.9% (Table 4). The predictive specificity was 95% and the area under curve (AUC) was 0.957 (Table 5).

**Table 4:** ROC curve analysis optimal cut value of CRMP4 methylation prediction PMLP

Criterion	Sensitivity	95% CI	Specificity	95% CI	Sensitivity + specificity
≥ 13.2	100	69.2–100.0	0	0.0–16.8	100
> 30.1	90.0	55.5–99.7	70.0	45.7–88.1	160.0
> 39.4	90.0	55.5–99.7	85.0	62.1–96.8	175.0
> *40.9*	*90.0*	*55.5–99.7*	*95.0*	*75.1–99.9*	*185.0*
> 41.4	80.0	44.4–97.5	95.0	75.1–99.9	175.0
> 42	70.0	34.8–93.3	100	83.2–100.0	170.0
> 44.8	30.0	6.7–65.2	100	83.2–100.0	130.0
> 48.6	0	0.0–30.8	100	83.2–100.0	100

**Table 5:** D-S-C-F test multiple comparisons between the two groups

Group	Wilcoxon Z	DSCF-value	P value
PMLP vs LPCa	3.74	5.29	< 0.001
PMLP vs LPCa-LN (−)	3.78	5.35	< 0.001
PMLP vs mPCa	3.33	4.70	0.01
PMLP vs mPCa-LN (+)	3.78	5.35	< 0.001
LPCa vs LPCa-LN (−)	3.78	5.35	< 0.001
LPCa vs mPCa	− 1.74	2.46	0.41
LPCa vs LPCa-LN (+)	1.06	1.50	0.83
LPCa-LN (−) vs mPCa	− 3.78	5.35	< 0.001
LPCa-LN (−) vs LPCa-LN (+)	− 3.78	5.35	< 0.001
mPCa vs LPCa-LN (+)	1.36	1.93	0.65

## Discussion

Primary malignant lymphoma prostate is an extremely rare disease and this disease does not have systemic symptoms [1, 14]. Although the definition of PMLP has been recommended by Bostwick and Mann, the diagnosis of PMLP remains suspect by some investigators [15]. Frequency and urgency are likely the most common symptoms. Currently, routine examination methods include cystoscopy, abdominal and pelvic ultrasound, CT, MRI, PET-CT, global bone scan, bone marrow biopsy, PSA, and LDH examination [14, 16, 17]. However, for such patients, PSA is not high, LDH is not completely increased, and imaging-specific findings are few. The vast majority of patients come into treatment due to urgency, frequent urination, increased nocturia, dysuria, acute urinary retention and other symptoms, whereas lymphoma-related symptoms, such as fever, night sweats and weight loss and other early systemic symptoms rarely occur. Thus, the clinical symptoms of PMLP are difficult to distinguish with symptoms of benign prostatic hyperplasia, prostate cancer or prostatitis. Furthermore, PMLP and prostate secondary lymphoma cannot be identified by morphology and IHC and must be combined with clinical manifestations, imaging data and lymph node and bone marrow biopsies to comprehensively diagnose. Moreover, the identification of PMLP and prostate secondary lymphoma is also very important. Therefore, developing more accurate and efficient methods to diagnose PMLP is urgent and important. DLBCL accounts for approximately 40% of B cell lymphoma, which is the largest subset of the lymphoma classification. T cell lymphoma is mainly seen in secondary prostate lymphoma [10, 18]. In this study, 10 cases of primary prostate lymphoma were B-cell lymphoma, which was consistent with the existing literature. DLBCL accounted for 90%, which is different from the DLBCL/PMLP ratio reported in the related literature [1]. The previous literature reported that DLBCL accounted for approximately 60% of PMLP [1]. The difference may be related to regional differences or a smaller sample size. The morphological characteristics of PMLP are the same as those occurring in the lymph nodes or other parts of the lymphoma. DLBCL can diffusely infiltrate the prostate tissue with sheets of large cell or B-cell phenotypes [19, 20]. Additionally, DLBCL may have pleomorphism and mitosis in common [20]. Mantle cell lymphoma (MCL) mainly consists of small to medium lymphocytes, manifesting as fuzzy nodules, diffuse, or rare nodular growth mode, and an irregular nucleus. One of the MCLs collected in this study exhibited a fuzzy nodule. Immunohistochemical staining plays important roles in the diagnosis and typing of lymphoma. CK, CD3, CD21, CD23, CD10, Bcl6, MUM1, Blc2, CD5, CyclinD1 and Ki-67 are the most common immunohistochemical combinations for prostate lymphoma. CK negative can rule out poorly differentiated prostate cancer. DLBCL expressing CD20 and Blc2 can be divided into germinal center subtype and non-germinal center subtype according to the expression of CD10, Bcl6 and MUM1. CD20, CD5, and CyclinD1 positive is the basis for the diagnosis of mantle cell tumor, and 1 case is positively expressed by these markers.

Our previous study found that CRMP4 promoter sites − 848, − 841, − 680, − 678, − 674, − 671, − 665, − 660, and − 658 loci are significantly methylated in PCa cell lines (PC-3, PC-3M, and DU-145) and mPCa (primary and metastatic lymph nodes) [21]. It was also confirmed that methylation of the CRMP4 promoter region inhibited the expression of CRMP4 in mPCa. This study retrospectively analyzed the clinical and pathological features of 10 cases of PMLP, and found that CRMP4 was significantly hypermethylated in PMLP tissue. The difference in CRMP4 methylation between PMLP and the other groups was significant, which suggested that CRMP4 methylation was a potential predictive index for PMLP. The difference of CRMP4 methylation values between the LPCa group, LPCa group and mPCa-LN (+) group, and mPCa group and mPCa-LN (+) group were not significant. Another study from our research group reported that the sensitivity and specificity of predicting prostate cancer lymph node metastasis is greater than 90% when the CRMP4 methylation value of prostate adenocarcinoma puncture tissue ≥ 15% [21]. The difference in CRMP4 methylation between the LPCa group and mPCa group is significant [21]. This may be related to the smaller number of cases collected in this study. We will continue to collect relevant information about PMLP patients in subsequent studies and obtain more reliable scientific data.

## Conclusion

Herein, to investigate the clinicopathological features of PMLP and the significance of CRMP4 methylation in PMLP, the methylation of CRMP4 promotor in each group [10 PMLP patients, 20 cases of prostate adenocarcinoma tissue, 10 cases LPCa-LN (−) and 10 cases mPCa-LN (+)] was analyzed statistically. We used ROC curve to analyze the diagnostic value of CRMP4 methylation in PMLP. The methylation of CRMP4 gene was significantly increased in PMLP, which is expected to become a new predictor for PMLP. The method had 90% sensitivity, 95% specificity under CRMP4 methylation > 40.9% condition.

## Abbreviations

PMLP: primary malignant lymphoma prostate
CRMP4: collapsin response mediator protein 4
LDH: lactate dehydrogenase
PSA: prostate specific antigen
LPCa: limited prostate cancer
HL: Hodgkin’s lymphoma
NHL: non-Hodgkin’s lymphoma
PCa: prostate cancer
IHC: immunohistochemistry
